# Concurrent Germline and Somatic Mutations in FLCN and Preliminary Exploration of Its Function: A Case Report

**DOI:** 10.3389/fonc.2022.877470

**Published:** 2022-05-19

**Authors:** Tao Wang, Yang Yang, Huayi Feng, Bo Cui, Zheng Lv, Wenlei Zhao, Xiangyi Zhang, Xin Ma

**Affiliations:** ^1^Department of Urology, The Third Medical Centre, Chinese People’s Liberation Army (PLA) General Hospital, Beijing, China; ^2^Medical School of Chinese People’s Liberation Army (PLA), Beijing, China

**Keywords:** Birt-Hogg-Dube (BHD) syndrome, folliculin (FLCN), mutation, renal cancer, TFEB/TFE3

## Abstract

Birt–Hogg–Dube syndrome is an autosomal dominant condition that arises from germline folliculin (FLCN) mutations. It is characterized by skin fibrofolliculomas, lung cysts, pneumothorax, and renal cancer. Here, we present the case of a 36-year-old woman with asymptomatic, multiple renal tumors and a history of spontaneous pneumothorax. Genetic analysis revealed a hotspot FLCN germline mutation, c.1285dupC (p.H429fs), and a novel somatic mutation, c.470delT (p.F157fs). This information and the results of immunohistochemical analysis of the renal tumors indicated features compatible with a tumor suppressor role of FLCN. Two transcription factors, oncogenic TFEB and TFE3, were shown to be regulated by FLCN inactivation, which results in their nuclear localization. We showed that a deficiency in the tumor suppressor FLCN leads to deregulation of the mammalian target of rapamycin signaling (mTOR) pathway. A potential link between FLCN mutation and ciliary length was also examined. Thus, the mutation identified in our patient provides novel insights into the relationship among FLCN mutations, TFEB/TFE3, mTOR, and cilia. However, an in-depth understanding of the role of folliculin in the molecular pathogenesis of renal cancer requires further study.

## Introduction

Birt–Hogg–Dube (BHD) syndrome is characterized by the development of fibrofolliculomas, lung cysts, and renal carcinoma. It is caused by germline mutations in the folliculin (FLCN) gene (1), a tumor suppressor gene that has been mapped to chromosome 17p11.2 (2). FLCN is currently the only gene associated with BHD. The spectrum of FLCN mutations has been outlined in several reports and summarized in a database (http://www.lovd.nl/flcn) (3). A hypermutable hotspot has been localized to a mononucleotide tract of eight cytosines within exon 11, and the most frequently observed mutation is the cytosine insertion c.1285dupC (2, 4).

The energy-sensing mammalian target of rapamycin (mTOR) pathway has been implicated in the pathogenesis of BHD (5, 6). The loss of functional folliculin caused by genetic mutations of FLCN may activate mTOR signalling that leads to the development of symptoms of BHD syndrome (6). TFEB and TFE3 are crucial transcription factors belonging to the MiTF/TFE family. FLCN plays a crucial role in mTORC1-mediated phosphorylation of TFEB (7). TFEB is activated by its nuclear localization, which occurs as a result of FLCN inactivation. The latter is correlated with reduced phosphorylation of TFEB. Overexpression of TFEB leads to kidney cysts and renal cell carcinoma (RCC) (8–10). FLCN inactivation also induces the transcriptional activity of TFE3 by promoting its nuclear localization and increased TFE3 activity is likely to be important for renal tumor development (11).

In their non-cycling resting state, most eukaryotic cells possess microtubule-based membranous protrusions from the cell surface referred to as primary cilia (12). FLCN is essential for ciliary localization and regulates mTOR signaling through primary cilia (13). Single point mutations within FLCN may disrupt these functions and cause cilia-related diseases (14).

In a sequencing analysis, we identified a hotspot germline variant of FLCN. We also confirmed a novel somatic mutation in tumor tissue that may confer additional familial risks. In the present study, we examined the associations among FLCN mutations, TFEB/TFE3, mTOR and cilia in a patient.

## Materials and Methods

### Immunohistochemistry (IHC) Staining

Tissue microarray were collected from 210 clear cell RCC (ccRCC) and 75 papillary RCC (pRCC) patients who underwent surgery at the Urology Department of the Chinese People’s Liberation Army (PLA) General Hospital (Beijing, China) from January 2012 to December 2020. This study was approved by the ethics committee of the Chinese PLA General Hospital.

The standard IHC staining protocols were followed as previously described (15). Slides were scanned using Axio Image Z2 Microscope (Zeiss) and TissueFAXS imaging system (TissueGnostics GmbH, Austria). The information of antibodies was listed in **Supplementary Table 3**.

### Immunofluorescence Staining

Tissue sections were first subjected to gradient dehydration and washed three times with PBS, fixed with 4% paraformaldehyde for 15 min, permeabilized with 0.5% Triton X-100, and then blocked with 5% goat serum for 30 min. Tissues were stained with primary antibodies (ARL13B, proteintech, 17711-1-AP) at 37°C for 1h and were incubated with AlexaFluor488-conjugated secondary antibodies (1:400). Nuclei were counterstained by 0.2 mg/mL DAPI. Samples were imaged with an Axio Image Z2 fluorescence microscope (Zeiss) and analyzed by using Image J (NIH) software to determine the percentage of ciliated cells, cilium length, and fluorescent signal intensity. The information of antibodies was listed in **Supplementary Table 3**. Prior to manually counting cilia, parameters were established by which putative cilia were to be included or excluded: 1) size; the cilium needed to be a thin structure, intensely stained broad structures were considered background; 2) elongation; the cilium needed to be a continuous thin extending structure, square or dot-like structures were excluded. Cilia is considered positive when the cilia ratio is greater than 1%.

### Sequencing

Genomic DNA was isolated using the Genomic DNA purification kit from Gentra Systems (Minneapolis, MN, USA). According to the manufacturer’s recommendations. Whole exome sequencing (WES) of genomic DNA from cancerous tissue was performed after the patient had provided informed consent, with effective sequencing depth of 300x.We then verified it with sanger sequencing and confirmed the germline mutation with paracancer tissue.

Paired samples of 3 papillary renal cell carcinomas and 3 clear cell carcinomas (cancer and paracancer) were used for control. The tumor tissues of these 6 patients were sequenced in the next generation, and no FLCN mutations were found.

## Case Presentation

**Figure 1:**
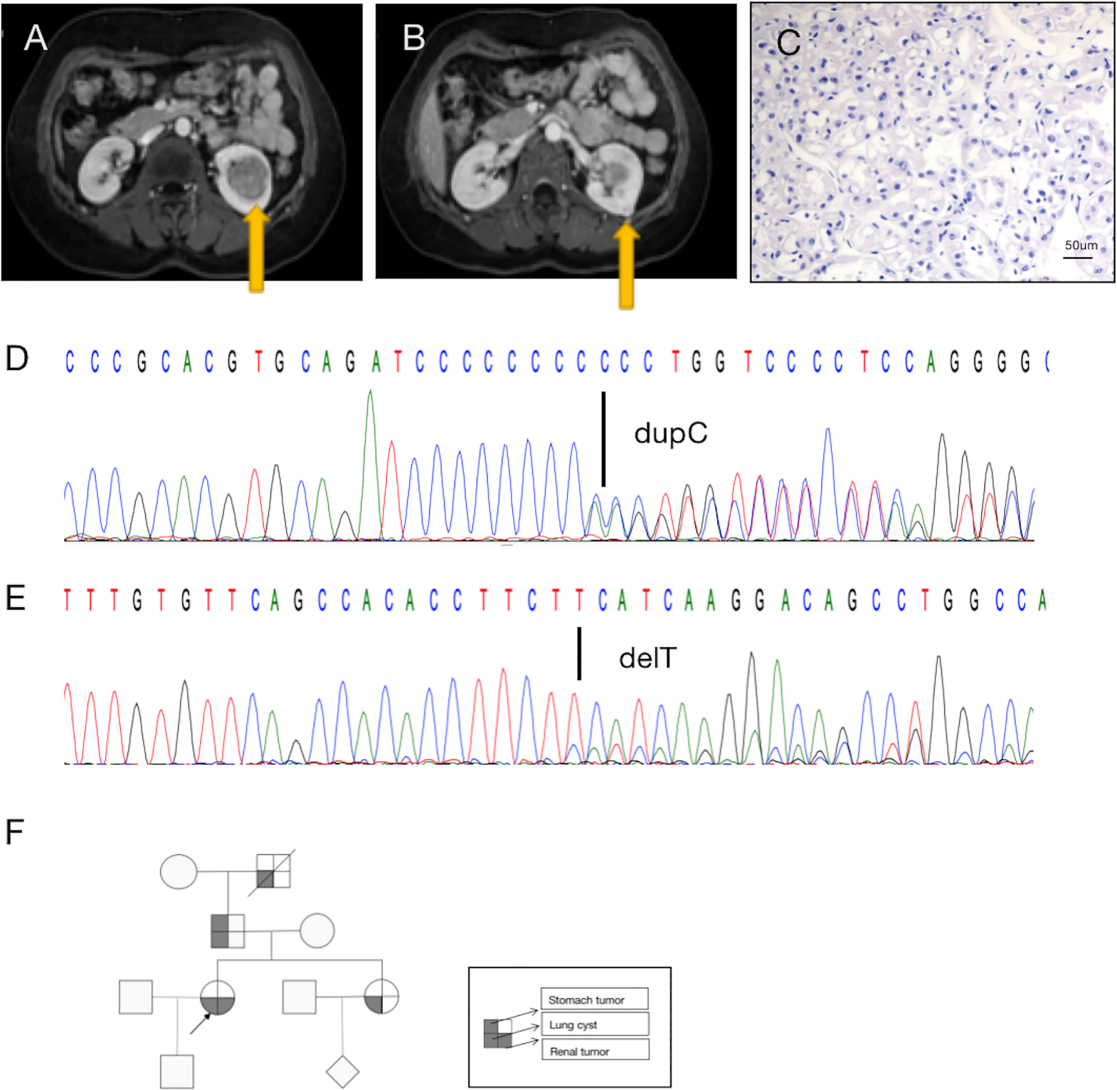
A diagnosis of BDH syndrome was confirmed in the patient. **(A)** Abdominal magnetic resonance imaging (MRI) revealed a renal mass in the lower middle part of the left kidney (arrow). **(B)** MRI with intravenous contrast revealed a renal mass in the posterolateral surface of the left kidney (arrow). **(C)** Histopathology of the small tumor in the left kidney shows cells with classic plant-like architecture, perinuclear clearing (chromophobe cell carcinoma). **(D)** Germline mutation: base C duplication at nucleotide c.1285dupC in exon 11 (c.1285dupC) of the FLCN gene. **(E)** Somatic mutation: a base T deletion at nucleotide c.470 in exon 6 (c.470delT) of the FLCN gene. **(F)** Pedigree summarizing the family history of the proband (arrowhead). Different symbols indicate different diseases.

A 36-year-old woman underwent ultrasonography and magnetic resonance imaging, which revealed multiple, asymptomatic renal tumors (**Figures 1A, B**). No apparent cutaneous lesions were found by careful inspection and palpation of the skin.

Retroperitoneal laparoscopic radical resection of the left kidney was performed with the patient under general anesthesia. Histopathologic examination (H&E staining) revealed that the large (3.5 cm × 3.5 cm × 3 cm) tumor in the left kidney was clear-cell RCC(image not available), nuclear grade WHO/ISUP II, and that the small (1.0 cm × 1.0 cm × 0.6 cm) tumor in the left kidney was chromophobe RCC, nuclear grade WHO/ISUP II (**Figure 1C**). In summary, the patient’s TNM stage was T1aN0M0, stage I. Due to limited sampling conditions, we only sequenced and analyzed the small tumor in the left kidney.

A search for germline mutations revealed a single nucleotide frameshift duplication, c.1285dupC, within the polycytosine tract located in exon 11, resulting in a change in amino acid 429 from histidine to proline (p.H429fs) in the FLCN product (**Figure 1D**). A search for somatic mutations revealed a base (T) deletion at nucleotide c.470 in exon 6 (c.470delT) of the FLCN gene (**Figure 1E**), which caused a frameshift mutation starting at amino acid 157 (p.F157fs). These results were consistent with a diagnosis of BHD.

In addition to the renal tumor, the patient had a history of spontaneous pneumothorax. Her family history was notable, as her sister, father, and grandfather had been diagnosed with lung cysts. No other family members had found skin abnormalities despite not being examined by a professional dermatologist. And they did not be diagnosed kidney tumors during routine physical imaging examination. There was a history of benign tumor of the cardia on the father’s side, and her grandfather had died of silicosis (**Figure 1F**). Unfortunately, the patient’s family members did not consent to genetic testing.

## Functional Analysis

**Figure 2:**
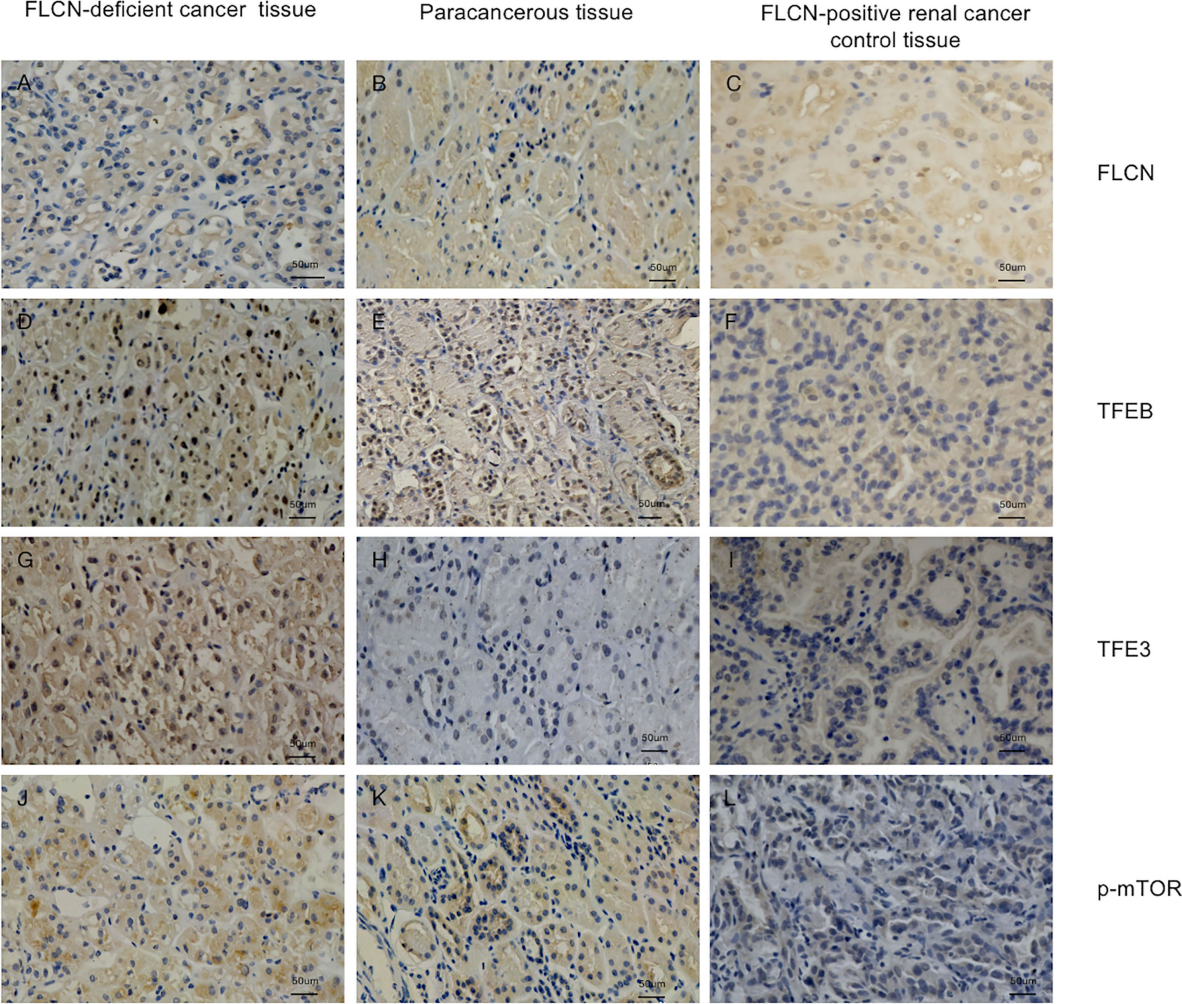
Immunostaining of the patient’s tumor tissue **(A, D, G, J)** and paracancerous tissue **(B, E, H, K)** and FLCN-positive control cancer tissue **(C, F, I, L)**. **(A–C)** Immunostaining of FLCN confirmed the loss of FLCN protein expression in patient-derived tumor tissue. **(D–F)** Immunostaining of TFEB confirmed that the protein was constitutively expressed in the nucleus and active in FLCN mutated tissue. **(G–I)** TFE3 was highly expressed in whole cells of tumor tissue, mainly in the nucleus. **(J–L)** p-mTOR was highly expressed in tumor and paracancer tissue, but negatively expressed in control tissues.

**Figure 3:**
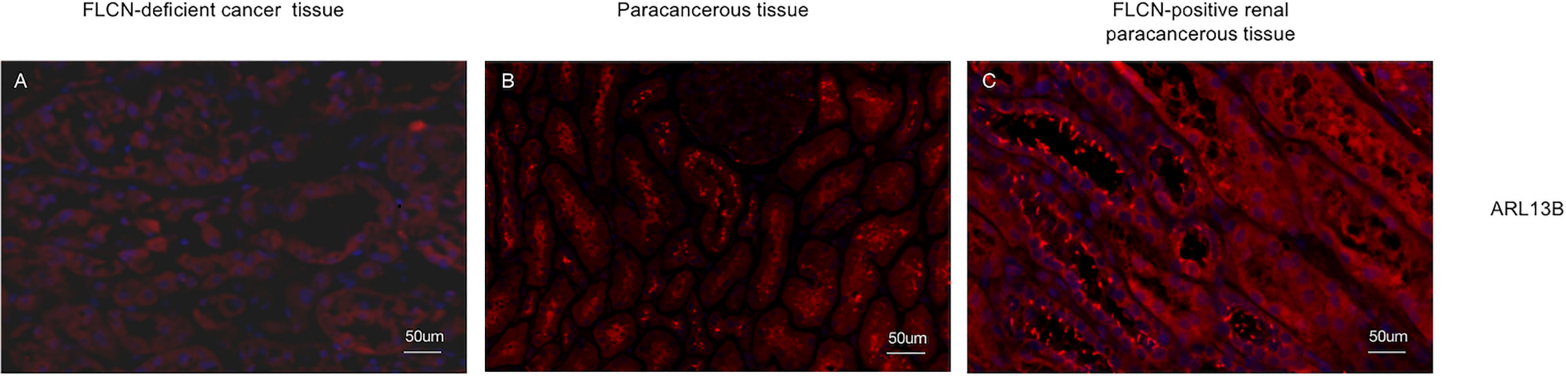
The cilia are rarely expressed in tumor tissue **(A)** and cilia length was reduced in the adjacent tissue **(B)** and cilia were normally present in the tissues without the FLCN mutation **(C)**.

To determine the function of the FLCN protein, immunohistochemistry/immunofluorescence staining was performed both on the patient’s tumor and adjacent tissues and on renal tissues from patients without FLCN mutations. FLCN immunostaining was markedly weaker in the renal tumor and adjacent tissue than in tissues without FLCN mutations (**Figures 2A–C**). TFEB was constitutively expressed in the nucleus and was active in tissues carrying the FLCN mutation (**Figures 2D–F**). TFE3 was highly expressed in whole cells of the FLCN-deficient tumor tissue, mainly in the nucleus, was weakly expressed in the adjacent tissue, and was not expressed in non-mutated tissues (**Figures 2G–I**). Phosphorylated mTOR (p-mTOR) was highly expressed in FLCN-deficient cancer and adjacent tissue, and negative in control tissues (**Figures 2K–L**). The cilia are rarely expressed in FLCN-deficient cancer tissue and cilia length was reduced in the adjacent tissue. By contrast, cilia were normally present in the tissues without the FLCN mutation(**Figures 3A–C**).

## Genetic Analysis

Somatic mutations in the ERF gene were also detected (exon4 c.911_913delCCT p.S304del). The mutation abundance is 6.4%. The **Supplementary Table 1** provides additional information that we sequenced for filtering out variations in genes with frequencies greater than 1% of a thousand genomes, variations with synonymous mutations, and variations that are benign and potentially benign.

## Discussion

Genetic studies have revealed several tumor suppressor genes responsible for the development of RCC, such as VHL, FH, TSC, SDHB, and FLCN, the focus of this study (16). Sequencing analysis revealed a hotspot germline variant of FLCN in our patient. We also identified a novel somatic mutation that merits attention as it may confer additional risks to family members.

Chromophobe RCC and a mixed pattern of chromophobe and oncocytic renal tumors are typical of patients with BHD, but RCCs with other histological subtypes can also occur (17). In our patient, H&E staining revealed that the large mass was ccRCC and the small mass chromophobe RCC. The presence of renal masses of different pathological types may suggest a relationship between the mutation hotspot and the pathology, but this awaits confirmation.

Normally, TFEB is located in the cytoplasm, and its phosphorylation by mTOR inhibits its function (18, 19). When FLCN is mutated, TFEB is dephosphorylated and freely enters the nucleus, where it participates in the transcription of its target genes (7). mTOR hyperactivity induced by TFEB is a key step in kidney cystogenesis and tumorigenesis. Recently, Ballabio and colleagues confirmed that TFEB promotes mTORC1 activity by transcriptionally regulating the levels of RagC and RagD GTPases (18). This sequence of events was consistent with our immunohistochemical results. Hong SB et al. (11) reported that FLCN inactivation was correlated with the post-translational modification and nuclear accumulation of TFE3. However, TFE3 was demonstrated by immunohistochemistry to be strongly expressed in cancer tissues but only weakly expressed in adjacent tissues in our data, which may due to the complexity of the tumor microenvironment.

Several studies have proposed that BHD is a novel ciliopathy (13, 14, 20). Single point mutations within FLCN can disrupt its ciliary location and cause cilia-related diseases (14). Basten et al. (21) evaluated renal tumor tissue cores from 110 RCC patients by immunofluorescent staining of cilia and showed reduced cilia frequency in RCC subtypes relative to adjacent non-tumor tissue suggesting that ciliary loss is common in renal cancer generally. Our study also supports this conclusion. Cilia staining was performed positive in 67/210 patients with clear cell RCC tissues, 35/75 patients with papillary RCC tissues, 274/285 patients with adjacent non-tumor tissues (**Supplementary Table 2**; **Supplementary Figure 1**). Besides, we discovered an interesting phenomenon in the patient: the cilia were abnormally shaped, forming short rods in the adjacent tissues, although it showed normal abundance. Normal cilia were found in tissues without FLCN mutations. This suggests that mutations in FLCN may affect the normal level and morphology of cilia. However, whether this is an accidental discovery or a general rule still requires large findings and mechanism exploration.

## Conclusions

In conclusion, the case is presented from a clinical perspective, providing personal and family history of BHD manifestations, genetic results for FLCN gene testing, and a second hit FLCN mutation in the patient tumor. A hotspot FLCN germline mutation, c.1285dupC (p.H429fs), and a novel somatic mutation, c.470delT (p.F157fs) were identified in the case presented with renal tumors and spontaneous pneumothorax. We explored the link between FLCN mutation and TFE3/TFEB expression, mTOR and primary cilia dysfunction *in vivo*. However, the role of folliculin in the molecular pathogenesis of renal cancer awaits further clarification.

## Data Availability Statement

The datasets presented in this study can be found in online repositories. The names of the repository/repositories and accession number(s) can be found in the article/**Supplementary Material**.

## Ethics Statement

The studies involving human participants were reviewed and approved by The Chinese PLA General Hospital. The patients/participants provided their written informed consent to participate in this study.

## Author Contributions

TW and YY: performed the research and wrote the article. HF and ZL: performed the experiments and collected patient data. BC, WZ, and XZ: assisted with laboratory experiments and produced radiology images. XM: contributed to patient samples and treated patients. All authors contributed to the article and approved the submitted version.

## Funding

The present work was financially supported by the National Natural Science Foundation of China (No. 81900718 and No. 81970665) and Chinese Postdoctoral Science Foundation (No. BSH47933-JD).

## Conflict of Interest

The authors declare that the research was conducted in the absence of any commercial or financial relationships that could be construed as a potential conflict of interest.

## Publisher’s Note

All claims expressed in this article are solely those of the authors and do not necessarily represent those of their affiliated organizations, or those of the publisher, the editors and the reviewers. Any product that may be evaluated in this article, or claim that may be made by its manufacturer, is not guaranteed or endorsed by the publisher.

## Supplementary Material

Supplementary Figure 1 The loss of cilia in tumor tissues accounts for a large proportion either ccRCC or pRCC (data seen in **Supplementary Table 2**).
