# Supplementary figures and images for: Concurrent Germline and Somatic Mutations in FLCN and Preliminary Exploration of Its Function: A Case Report
Source: Front Oncol. 2022 May 19;12:877470. doi: 10.3389/fonc.2022.877470 (PMC9162506; doi:10.3389/fonc.2022.877470)

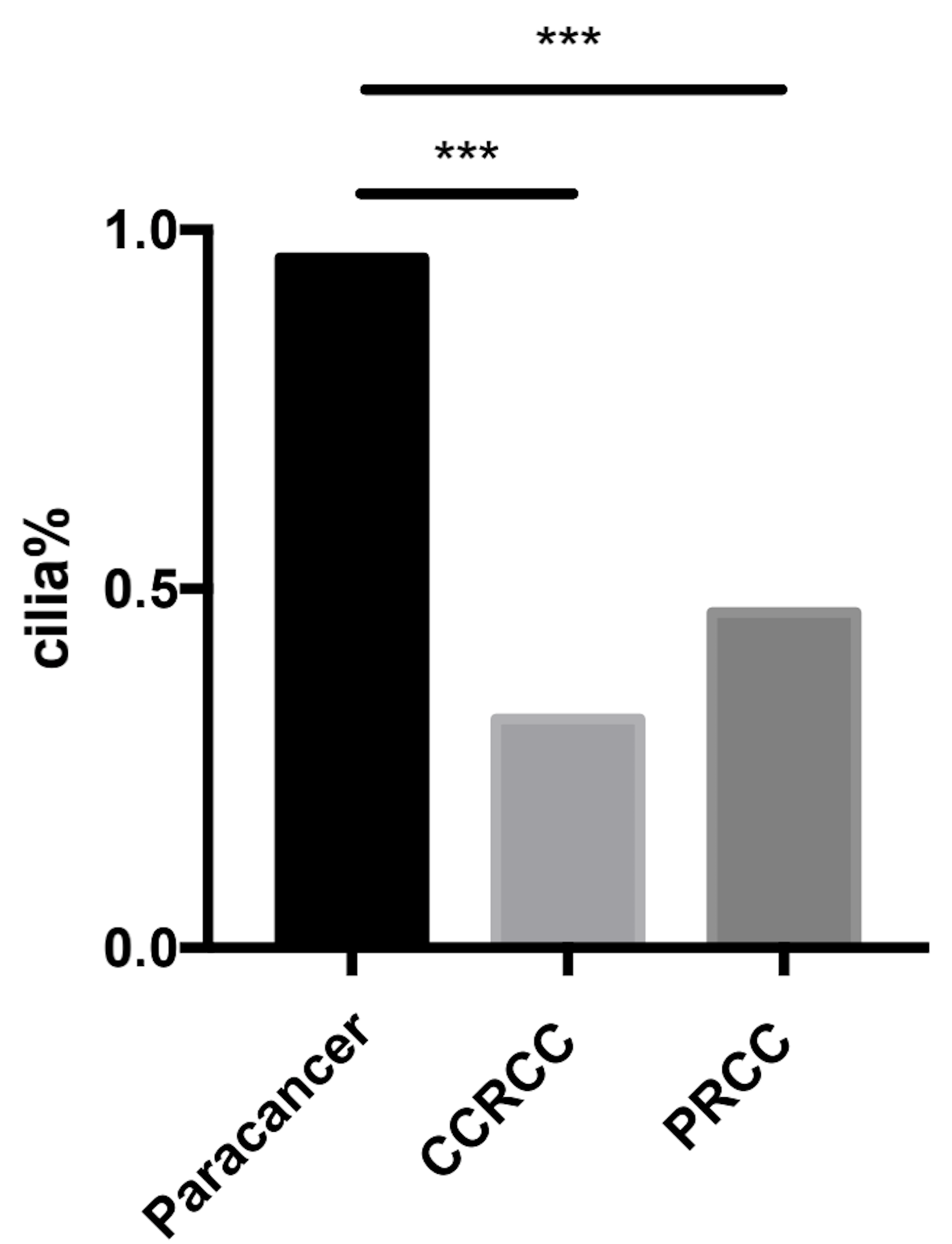

Supplement: Supplementary Figure 1 — The loss of cilia in tumor tissues accounts for a large proportion either ccRCC or pRCC (data seen in Supplementary Table 2). [file Image_1.jpeg]
