# Supplementary material for: Concurrent Germline and Somatic Mutations in FLCN and Preliminary Exploration of Its Function: A Case Report
Source: Front Oncol. 2022 May 19;12:877470. doi: 10.3389/fonc.2022.877470 (PMC9162506; doi:10.3389/fonc.2022.877470)
Supplement: Supplementary file 2 [file Table_1.docx]

**Supplementary Table** **1 |** Additional information of gene analysis.

| **Chromosome** | **Gene** | **Variant**  **(DNA level)** | **Variant**  **(protein level)** | **Effect** | **Abundance** |
| --- | --- | --- | --- | --- | --- |
| 4 | CRIPAK | c.487_517dupGACGTGGAGTGCCCGCCTGCTCACACGTGCC | p.P173fs | Frameshift | 98.10% |
| 17 | KRTAP9-6 | c.315_316delTGinsCT | p.G106C | missense | 95.90% |
| 11 | MUC2 | c.4678A>T | p.T1560S | missense | 63.00% |
| 3 | ARHGEF26 | c.178_179delCTinsTC | p.L60S | missense | 51.50% |
| 8 | ZC3H3 | c.2638_2639delTCinsAA | p.S880K | missense | 41.70% |
| 5 | PCDHGA1 | c.2048A>T | p.K683I | missense | 41.60% |
| 1 | TCHH | c.1105_1143dupGAGGAGGAGAGGCGCGAGCAGCAGCTGAGGCGCGAGCAG | p.Q381_Q382insE  EERREQQLRREQ | Non shift insertion | 39.00% |
| 3 | ERICH6 | c.138_158dupAGAGGTGGAGGAGGAGGAGGA | p.E53_E54insEVEEEEE | Non shift insertion | 38.80% |
| 17 | LOC100506388 | c.375_*52delAGGCCATTAGAGCTCACCACCCCGGGCGTTCGTCACTGC  AGACATCACTGCAGACACAGAGA | p.E125fs | Frameshift | 38.50% |
| 11 | DEUP1 | c.1318_1319delGAinsCG | p.E440R | missense | 32.30% |
| 21 | DONSON | c.1490C>T | p.S497F | missense | 20.40% |
