# Supplementary material for: Concurrent Germline and Somatic Mutations in FLCN and Preliminary Exploration of Its Function: A Case Report
Source: Front Oncol. 2022 May 19;12:877470. doi: 10.3389/fonc.2022.877470 (PMC9162506; doi:10.3389/fonc.2022.877470)
Supplement: Supplementary file 3 [file Table_2.docx]

|  | Paracancerous tissues | ccRCC tissues | pRCC tissues | χ2 /F value | P value |
| --- | --- | --- | --- | --- | --- |
| n | 285 | 210 | 75 | 236.540 | 0.000 |
| cilia | 274 | 67 | 35 |  |  |
| Without cilia | 11 | 143 | 40 |  |  |

**Supplementary table 2 |** The ratio of cilia.
