# Supplementary material for: Concurrent Germline and Somatic Mutations in FLCN and Preliminary Exploration of Its Function: A Case Report
Source: Front Oncol. 2022 May 19;12:877470. doi: 10.3389/fonc.2022.877470 (PMC9162506; doi:10.3389/fonc.2022.877470)
Supplement: Supplementary file 4 [file Table_3.docx]

| Name | Manufactures | Number | Type | Usage |
| --- | --- | --- | --- | --- |
| FLCN | Proteintech | 11236-2-AP | Polyclonal | IHC |
| TFEB | Proteintech | 13372-1-AP | Polyclonal | IHC |
| TFE3 | Proteintech | 14480-1-AP | Polyclonal | IHC |
| Phospho-MTOR (Ser2448) | Proteintech | 80596-1-RR | Recombinant Antibody | IHC |
| ARL13B | Proteintech | 17711-1-AP | Polyclonal | IF |

**Supplementary Table 3 |** Antibodies.
